# Cost-minimization analysis of oral versus intravenous antibiotic treatment for *Klebsiella pneumoniae* liver abscess

**DOI:** 10.1038/s41598-023-36530-5

**Published:** 2023-06-16

**Authors:** Joanne Yoong, Kah Hung Yuen, James S. Molton, Ying Ding, Boon Piang Cher, Monica Chan, Shirin Kalimuddin, Jolene Oon, Barnaby Young, Jenny Low, Brenda M. A. Salada, Tau Hong Lee, Li Min Wijaya, Dale Fisher, Ezlyn Izharuddin, Yuan Wei, Rachel Phillips, Rajesh Moorakonda, David C. Lye, Sophia Archuleta

**Affiliations:** 1 Research for Impact, Singapore, Singapore; 2 Dean’s Office, Yong Loo Lin School of Medicine, National University of Singapore, Singapore, Singapore; 3 Division of Infectious Diseases, University Medicine Cluster, National University Hospital, Singapore, Singapore; 4 Department of Medicine, Yong Loo Lin School of Medicine, National University of Singapore, Singapore, Singapore; 5 Infectious Diseases Department, Tan Tock Seng Hospital, Singapore, Singapore; 6 National Centre for Infectious Diseases, Singapore, Singapore; 7 Agency for Care Effectiveness, Singapore, Singapore; 8 Department of Infectious Diseases, Singapore General Hospital, Singapore, Singapore; 9 Duke-NUS Medical School, Singapore, Singapore; 10 Lee Kong Chian School of Medicine, Nanyang Technological University, Singapore, Singapore; 11 Singapore Clinical Research Institute, Singapore, Singapore; 12 School of Public Health, Imperial College London, London, UK

**Keywords:** Health care economics, Clinical trial design

## Abstract

A cost-minimization analysis was conducted for *Klebsiella pneumoniae* liver abscess (KLA) patients enrolled in a randomized controlled trial which found oral ciprofloxacin to be non-inferior to intravenous (IV) ceftriaxone in terms of clinical outcomes. Healthcare service utilization and cost data were obtained from medical records and estimated from self-reported patient surveys in a non-inferiority trial of oral ciprofloxacin versus IV ceftriaxone administered to 152 hospitalized adults with KLA in Singapore between November 2013 and October 2017. Total costs were evaluated by category and payer, and compared between oral and IV antibiotic groups over the trial period of 12 weeks. Among the subset of 139 patients for whom cost data were collected, average total cost over 12 weeks was $16,378 (95% CI, $14,620–$18,136) for the oral ciprofloxacin group and $20,569 (95% CI, $18,296–$22,842) for the IV ceftriaxone group, largely driven by lower average outpatient costs, as the average number of outpatient visits was halved for the oral ciprofloxacin group. There were no other statistically significant differences, either in inpatient costs or in other informal healthcare costs. Oral ciprofloxacin is less costly than IV ceftriaxone in the treatment of Klebsiella liver abscess, largely driven by reduced outpatient service costs.

Trial registration: ClinicalTrials.gov Identifier NCT01723150 (7/11/2012).

## Introduction

*Klebsiella pneumoniae* liver abscess (KLA) is an invasive liver abscess syndrome caused by hypervirulent strains of *Klebsiella pneumoniae* that may also be associated with metastatic infection to the eye, brain or lung^1,2^. While KLA emerged in East Asia, over recent decades it has been increasingly reported in other parts of the world including the United States^3–6^. To date, the optimal management of KLA has been unclear due to a paucity of rigorous clinical evidence. Following drainage, patients typically receive intravenous (IV) antibiotic treatment for up to 6 weeks^1,2^. However, IV treatment is more costly than oral therapy with agents such as ciprofloxacin and trimethoprim-sulfamethoxazole, and may also increase the risk of IV catheter complications^7^.

Recently, Molton et al. demonstrated that oral ciprofloxacin was clinically non-inferior to IV ceftriaxone for KLA, concluding that early switch to oral antibiotics did not compromise patient outcomes^8^. However, the question of cost-effectiveness of oral versus IV antibiotics in the case of KLA has not been investigated. While oral antibiotics themselves may potentially be less costly and intuitively should reduce the burden on healthcare systems, it is not known whether such treatment merely serves to transfer the economic burden, in the form of other costs such as productivity losses, to the patient and their family^9^.

To explore these cost implications of a shift to oral from IV antibiotics, a cost-minimization analysis comparing costs from the formal healthcare sector and societal perspectives was conducted as part of the Antibiotics for Klebsiella Liver Abscess (A-KLASS) trial^10^. The A-KLASS trial was a multi-center, open-label, parallel group, randomized clinical trial of early oral antibiotics versus continued IV antibiotics for KLA conducted at 3 academic medical centers in Singapore^8^.

## Methods

### Clinical procedures

As per the A-KLASS trial protocol, eligible patients were randomized 1:1 to either continue IV antibiotics (control group) or switch to oral antibiotics immediately following randomization (intervention group)^11^. The trial recruited hospitalized adults with liver abscess and *Klebsiella pneumoniae* isolated from blood or abscess fluid who had received ≤ 7 days of effective antibiotics at 3 sites in Singapore. All patients in the control group received IV ceftriaxone 2 g once daily, while all patients in the intervention group received oral ciprofloxacin 500–750 mg twice daily. Study drug was continued for 4 weeks post-randomization. All patients had baseline demographic and weekly clinical data recorded. Decisions regarding abscess drainage, mode of drainage, and timing of hospital discharge were at the treating doctor’s discretion.

On discharge, patients in the intervention group were dispensed oral antibiotics for self-administration. Patients in the control group either completed the IV antibiotics treatment in the hospital prior to discharge or had a peripherally inserted central catheter and received IV antibiotic via outpatient parenteral antibiotic therapy (OPAT) services. If at the week 4 visit patients met criteria for clinical response, antibiotics were discontinued. If these criteria were not met, oral antibiotics were administered in 2-weekly extensions with visits every 2 weeks and monthly imaging until clinical response. A final study visit was scheduled for all participants at week 12. These requirements were the same for both control and intervention groups. Both groups had additional outpatient visits as needed for clinical care. The types of visits included: OPAT visits for antibiotics (which included the cost of the infusion pump for the control IV group) laboratory tests with associated nurse & physician fees; radiology visits for repeat imaging; surgical follow-up visits for patients who required abscess drainage; other visits as needed for patient care.

The primary endpoint for efficacy was “clinical cure,” determined at week 12 and defined as CRP < 20 mg/L, plus temperature < 38 °C at the week 12 assessment and the preceding week, plus reduced maximal diameter of the abscess at the most recent abdominal imaging^8^.

Demographic and outcome data were collected for clinical comparison. In addition to the primary endpoint, this included all-cause mortality, unplanned re-admission, unplanned abscess drainage, metastatic complications, recurrent *K. pneumoniae* bacteremia, length of hospitalization, duration of medical leave following discharge, and proportion with antibiotic adherence ≥ 80%.

### Cost data

This study adopted the cost classification proposed by Neumann et al.^12^. Formal healthcare sector cost included inpatient and outpatient treatment costs, traditional diet/supplements paid out-of-pocket by patients and/or their families, related equipment and care from home healthcare professionals. Self-reported informal healthcare sector costs included transport, but did not include patient time to undergo diagnosis and healthcare services and therapy or unpaid caregiver time. Non-healthcare sector costs included productivity losses incurred by patients and caregivers, as well as the costs of other professional support services (primarily domestic home help in the Singapore context).

Formal healthcare costs were estimated using inpatient and outpatient administrative billing records for all visits reported at the study sites extracted for all patients over the study period. The daily cost of antibiotics was $1.110 for the control group, and $0.240–0.378 depending on the dose used for the intervention group. There was no difference in charges to trial participants versus non-trial patients with KLA. Only urine pregnancy testing, glucose-6-phosphate dehydrogenase assays, procalcitonin and blood collected for host studies, which were not considered standard of care, were covered by the study grant. Total charges were used as a proxy for hospital costs using a top-down approach, net of procedures and tests conducted only for study purposes.

Singapore follows a mixed healthcare financing system with multiple payers, under which public hospitals are directly funded by the government using global budgets as well as prospective partial reimbursement based on diagnosis-related groups for inpatient and day-surgery services and per piece rates for outpatient visits. The public health insurance system, MediShield, covers inpatient and selected outpatient services. Premiums, deductibles, co-insurance, co-payments and costs above the claim limit may be paid out of balances held in nationally-mandated individual Medisave health savings accounts. Individuals may also elect to purchase supplemental private insurance coverage, or be covered by employer health insurance. The remaining expenditures are paid for out-of-pocket, with a number of means-tested supports for the low-income individuals including a general endowment fund, MediFund and other more targeted individual subsidy schemes^13^.

Out-of-pocket transport expenses related to informal healthcare and productivity losses were collected via patient surveys at week 12 follow-up. Transportation costs included expenses incurred by the patient and anyone who accompanied the patient. Average self-reported transport costs for inpatient and outpatient visits were assumed to apply both ways to each visit.

Productivity losses refer to the economic impact of reduced work productivity experienced by the patients or their caregivers. These economic losses stem from absence or resignation from paid employment. For individuals in paid employment, the losses were valued using the human capital approach^10,14^ by multiplying self-reported hours of work lost by self-reported wages for individuals and caregivers based on current gross salary during the 12-week trial; for those who resigned, the losses were valued based on their last drawn gross salary for the period between their last day at work and the end of the trial. Wages provided in monthly or weekly terms were converted to hourly equivalents using average hours worked per week and days worked per month for Singapore based on Ministry of Manpower statistics^15^.

Costs were denominated in 2018 Singapore Dollars (SGD), at the exchange rate of 1 SGD = 0.74 USD^16^. All costs were reported cumulatively over the study period and inflated to 2018 prices, which corresponds to the end year of the study, using the healthcare component of the annual Consumer Price Index (CPI)^16^ and weighted by the relative time spent in each calendar year. Total societal costs were computed by summing the formal healthcare sector costs, informal healthcare costs, and productivity losses^12^.

### Statistical analysis

Statistical analysis was performed using Stata®16^17^. Descriptive statistics were presented in the form of means, medians and the interquartile range. Differences in means between groups were compared using an independent samples two-tailed *t* test for unequal variances. Categorical variables were analyzed using Fisher’s exact test. Statistical significance was defined by a *p* value of < 0.05.

The baseline uses a complete case analysis (Tables 3, 4, 5, 6), whereas the sensitivity analysis uses an available case analysis for each constituent cost (i.e., formal, informal, and non-healthcare sector costs) (Table 7). Missing values were not imputed as they were relatively few.

### Ethics approval

The trial was approved by the National Healthcare Group Domain Specific Review Board (2012/01035) and Centralised Institutional Review Board (2013/747/F). Written informed consent was obtained from all subjects and/or their legal guardian(s). The study was conducted in accordance with the Declaration of Helsinki.

## Results

Of 152 patients randomized, 71/74 (95.9%) randomized to oral antibiotics met the primary endpoint of clinical cure, compared with 72/78 (92.3%) randomized to IV antibiotics (risk difference, 3.6%; 2-sided 95% confidence interval, − 4.9% to 12.8%) thus meeting the pre-defined margin of non-inferiority. Of these, cost data was collected from 139 patients. Table 1 shows the number of subjects recruited across the centers and the assignment to the control group (IV antibiotics) and intervention group (oral antibiotics). Table 2 shows the patient demographics and comparative use of antibiotics overall.

**Table 1:** Recruitment summary.

Recruiting centers	IV ceftriaxone (n = 69)	Oral ciprofloxacin (n = 70)
NUH	30	30
SGH	13	12
TTSH	26	28

**Table 2:** Patient demographics.

Characteristics, n (%)	IV ceftriaxone (n = 69)	Oral ciprofloxacin (n = 70)	All subjects (n = 139)
Age			
Mean (SD)	56.4 (13.0)	60.1 (14.5)	58.3 (13.8)
Median (Q1–Q3)	58 (44–72)	62 (45–79)	58 (42–74)
Gender (n, %)			
Male	53 (76.8)	52 (74.3)	105 (75.5)
Female	16 (23.2)	18(25.7)	34 (24.5)
Ethnicity (n, %)			
Chinese	52 (75.4)	58 (82.9)	110 (79.1)
Indian	0 (0.0)	0 (0.0)	0 (0.0)
Malay	16 (23.2)	10(14.3)	26 (18.7)
Other	1 (1.4)	2 (2.9)	3 (2.2)
Medical condition (n, %)			
Diabetes mellitus	38 (55.1)	30 (42.9)	68 (48.9)
Cardiovascular disease	27 (39.1)	26 (37.1)	53 (38.1)
Pulmonary disease	4 (5.8)	6 (8.6)	10 (7.2)
Hepatic disease	4 (5.8)	5 (7.1)	9 (6.5)
Biliary tract disease	8 (11.6)	4 (5.7)	12 (8.6)
Gastrointestinal tract disease	5 (7.3)	7 (10.0)	12 (8.6)
Chronic kidney disease	1 (1.5)	3 (4.3)	4 (2.9)
Malignancy	2 (2.9)	4 (5.7)	6 (4.3)
Neurologic disease	5 (7.3)	3 (4.3)	8 (5.8)
Rheumatologic/autoimmune disease	2 (2.9)	0 (0.0)	2 (1.4)
Others	45 (65.2)	44 (62.9)	89 (64.0)
Antibiotics			
Number of antibiotics since admission (mean, SD)	2.8 (1.2)	2.9 (1.1)	2.8 (1.1)
Number of antibiotics since admission (median, Q1–Q3)	3.0 (1.0–5.0)	3.0 (1.0–5.0)	3.0 (1.0–5.0)
Patients with effective antibiotic use (n, %)	70 (100.0)	69 (100.0)	139 (100.0)
Days of effective antibiotics, Median (Q1–Q3)	5.0 (3.0–7.0)	5.0 (4.0–6.0)	5.0 (3.0–7.0)

### Formal healthcare sector costs

Table 3 shows the formal healthcare sector costs over the 12-week trial period. The mean total formal healthcare sector cost for the oral antibiotic group was $15,013, which was $3772 (or 20%) lower than the IV antibiotic group’s $18,785 (*p* = 0.008). Relative cost savings were primarily/largely driven by lower outpatient costs in the oral antibiotic group ($3596 vs. $2280, *p* < 0.001). Although inpatient cost constituted the majority of the total formal healthcare cost for both groups, there was no statistically significant difference ($15,171 vs. $12,680, *p* = 0.08). Very few patients incurred expenses for other healthcare sector costs such as durable medical equipment or supplementary medicine/services. The costs incurred for these two components were not statistically different for the two groups at the mean.

**Table 3:** Formal healthcare sector costs (per patient).

	IV ceftriaxone (n = 62)	Oral ciprofloxacin (n = 66)	Overall	*T* test/Fisher’s exact test *P* value
Total formal healthcare sector costs	$18,785	$15,013	$16,840	0.008
Outpatient services	$3596	$2280	$2917	< 0.001
Inpatient stay	$15,171	$12,680	$13,886	0.08
Durable medical equipment	$6	$15	$11	0.26
No. (%) of patients who incurred out-of-pocket expenses for durable medical equipment	3 (5%)	6 (9%)	9 (7%)	0.49
Supplementary medicine/services	$13	$39	$26	0.11
No. (%) of patients who incurred out-of-pocket expenses for supplementary medicine/services	8 (13%)	11 (17%)	19 (15%)	0.62

### Informal healthcare sector costs

Table 4 shows the mean informal healthcare sector costs per patient over the 12-week trial. Transport expenses were incurred by most patients and any accompanying individuals barring a small number of missing responses. Compared with the IV antibiotic group, the oral antibiotic group saved an average of $36 (or 21%) on transport although the difference was not statistically significant ($172 vs. $136, *p* = 0.29). Visits to healthcare institutions for outpatient care made up the bulk of the transport cost for both groups. Although the oral antibiotic group had half the number of outpatient visits compared with the IV antibiotic group (19 vs. 9, *p* < 0.001), the reduction in transport expenses was proportionately smaller (28%) and not statistically significant ($159 vs. $115, *p* = 0.18). Corresponding to the low number of all-cause inpatient re-admissions per patient for the IV and oral antibiotic groups (0.3 vs. 0.4, *p* = 0.10), both groups incurred minimal transport expenses for inpatient care and the difference was both small in magnitude and statistically insignificant ($13 vs. $21, *p* = 0.46).

**Table 4:** Informal healthcare sector costs (mean per patient).

	IV ceftriaxone (n = 62)	Oral ciprofloxacin (n = 66)	Overall	*T* test *P* value
Total informal healthcare sector costs (transport expense)	$172	$136	$153	0.29
Transport expense for outpatient visits	$159	$115	$136	0.18
Transport expense for inpatient re-admissions	$13	$21	$17	0.46

#### Non-healthcare sector costs

Table 5 shows the non-healthcare sector costs. The total non-healthcare sector cost of the oral antibiotic group was $1229, which was $383 (or 24%) lower than the IV antibiotic group’s $1612 although the difference was not statistically significant (*p* = 0.45).

**Table 5:** Non-healthcare sector costs (mean per patient).

	IV ceftriaxone (n = 62)	Oral ciprofloxacin (n = 66)	Overall	*T* test /Fisher’s exact test *P* value
Total non-healthcare sector costs	$1612	$1229	$1415	0.45
Patient’s own productivity losses	$1535	$819	$1166	0.14
No. (%) of patients still employed	39 (63%)	31 (47%)	70 (55%)	0.08
Hours absent from work for patients still employed	187 h	111 h	153 h	0.12
No. of patients resigned	3	1	4	0.29
Monthly salary of patients who were still employed or had resigned	$3164	$3391	$3260	0.83
Caregiver productivity losses	$0	$267	$138	0.09
No. (%) of patients with caregivers	8 (13%)	22 (33%)	30 (23%)	0.007
No. (%) of caregivers in employment	4 (50%)	11 (48%)	15 (48%)	1.00
Hours absent from work for caregivers in employment	0 h	110 h	76 h	0.04
Monthly salary of caregivers in employment	$2600	$3589	$3409	0.35
Professional care support services/domestic helper	$77	$144	$112	0.64
No. (%) of patients who engaged professional care support services/domestic helper	1 (2%)	5 (8%)	6 (5%)	0.21

There was no statistically significant difference in productivity losses per patient across the groups ($1535 vs. $819, *p* = 0.14). About half of patients in our sample were employed for the entire duration of the study. Although work absences for those in the IV group exceeded those of the oral treatment group by 1.7 times, this difference was not statistically significant (187 h vs. 111 h, *p* = 0.12). Seven patients—a small number—reported spending extra hours to catch up on work and these were not included in the computation of productivity losses. Four patients resigned from paid employment, of which three left their jobs by the first inpatient admission while resignation dates were missing for the remaining one. Thus, the corresponding productivity losses for the four patients over the full 12 weeks—corresponding to the entire trial period—were estimated. Monthly salaries—current salary for those employed and last drawn salary for those who had resigned—were not significantly different between groups.

No statistically significant difference was found for the productivity losses from the caregivers of the patients ($0 vs. $267, *p* = 0.09). A quarter of all patients received caregiving, with half as many patients in the IV group having so compared with the oral treatment group (13% vs. 33%, *p* = 0.007). Apart from a patient in the oral treatment group with two caregivers, the rest of the patients who received caregiving had one caregiver each. Half of caregivers were employed, and none of the employed caregivers had resigned for caregiving reasons. Despite the limited number of observations (less than 15), employed caregivers of IV patients were not absent from work, whereas caregivers for the oral treatment group totaled an average absence of 110 h (*p* = 0.04). However, the difference in the monthly wages and the subsequently computed productivity loss of the caregivers did not reach statistical significance between the two groups.

Finally, few patients hired professional home help to support caregiving. These costs were also not statistically different between the two groups ($77 vs. $144, *p* = 0.64).

#### Societal cost

Table 6 presents the mean figures per patient over the trial period of 12 weeks for the 128 patients with complete data across all categories. The oral antibiotics group achieved societal cost savings of $4191 (or 20%), from $20,569 to $16,378. The result was strongly significant (*p* = 0.004) and driven by the reduction in formal medical costs of $3772 (*p* = 0.008). Cost savings from other sources were not significantly different between the two groups.

**Table 6:** Societal cost (mean per patient). 95% confidence intervals in parentheses.

	IV ceftriaxone (n = 62)	Oral ciprofloxacin (n = 66)	Overall	*T* test *P* value
Societal cost	$20,569 (18,296–22,842)	$16,378 (14,620–18,136)	$18,408 (16,954–19,862)	0.004
Formal healthcare sector costs	$18,785 (16,597–20,973)	$15,013 (13,292–16,734)	$16,840 (15,438–18,243)	0.008
Informal healthcare sector costs	$172 (113–231)	$136 (102–169)	$153 (120–186)	0.29
Non-healthcare sector costs	$1612 (809–2416)	$1229 (611–1848)	$1415 (918–1912)	0.45

### Sensitivity analysis

We subjected our baseline to three separate scenarios in the sensitivity analysis. First, we substituted baseline for healthcare CPI for the relevant year when computing informal healthcare and non-healthcare sector costs. Second, in examining the cost breakdown into eight subcategories, we applied the Bonferroni correction to explore robustness to controls for multiple hypothesis testing (implying a threshold of 0.625% for an alpha of 5%). Third, to minimize data loss, we recomputed Tables 3, 4 and 5 using available case analysis and reported the results in Table 7. We find that none of these scenarios significantly change the magnitude or significance of our findings.

**Table 7:** Sensitivity analysis using available case analysis.

	IV ceftriaxone	Oral ciprofloxacin	Overall	*T* test *P* value
Total formal healthcare sector costs	$19,074	$15,830	$17,441	0.03
Outpatient services	$3601	$2295	$2943	<0.001
Inpatient stay	$15,456	$13,485	$14,463	0.18
Durable medical equipment	$5	$14	$10	0.24
Supplementary medicine/services	$12	$36	$25	0.12
Total informal healthcare sector costs (transport expense)	$172	$135	$153	0.25
Transport expense for outpatient visits	$162	$114	$137	0.13
Transport expense for inpatient visits	$15	$24	$19	0.42
Total non-healthcare sector costs	$1538	$1200	$1364	0.49
Patient’s own productivity losses	$1464	$794	$1119	0.15
Caregiver productivity losses	$0	$252	$127	0.09
Productivity care support services/domestic helper	$69	$149	$109	0.55

## Discussion

In our study, we found that the use of oral rather than IV antibiotics for *Klebsiella pneumoniae* liver abscess demonstrated a significant societal cost reduction of over $4000 per patient over the 12-week treatment period without compromising clinical outcome, driven largely by reductions in formal healthcare sector costs. We find that although the oral regime suggests more caregiver involvement, relative to the existing standard of care, cost savings are significant and amount to about 20% of the mean total cost.

It is notable that the majority of patients in the IV treatment group were on outpatient parenteral antimicrobial therapy (OPAT), which has been shown to be relatively cost-effective^18^. However, our study finds that even lower costs (especially outpatient costs) may be achieved with the use of oral antibiotics. In the Singapore context, as these benefits are shared across different payers in the health system—these savings accrue to both the government (in the form of lower reimbursements) and patients (in the form of lower out-of-pocket expenses), which suggests that the incentives to shift to oral antibiotics are well-aligned across stakeholders.

While our results are particular to Singapore, these implications may be extended more broadly to health systems where outpatient services are not highly developed or readily accessible given the target population. For instance, in settings where antibiotics can only be delivered in a hospital or a nursing home, we anticipate that the length of stay and cost differences would be even greater.

Moreover, we note that the shift in modality to oral antibiotics significantly reduced the burden of outpatient visits, which in other environments may result in larger realized cost savings. Our study took place in a highly urban setting with generally healthy patients of early retirement age—typically already not working but ambulatory and not requiring significant caregiver assistance. As a result, in our setting, the reduction in outpatient visits resulted in relatively small realized transportation cost savings, and although considerable time was saved from the patient perspective, there were relatively few instances of averted work-loss for patients and caregivers. In other settings where travel costs are significant or where patients are of working-age or require caregiver support, we anticipate that productivity-related benefits may be of sizable value.

This study has several key limitations. Notably, we did not estimate the value of time to patients or caregivers. We are also constrained by a relatively small sample size, and hence lack the power to statistically detect cost differences which may be meaningful at scale. Given the considerable time savings under the oral modality as noted, this suggests that our results are likely to underestimate the true societal cost savings in favor of oral antibiotics. We conducted only a deterministic sensitivity analysis. Finally, our findings are limited to the study of one specific infection and two specific antibiotics. However, they may be relevant to other infections where oral antibiotics have been also shown to be non-inferior, including infective endocarditis^19^ and bone/joint infections^20^, and highlight the importance of including more economic evaluations alongside similar trials in the future.

## Data Availability

The datasets generated during and/or analyzed during the current study are available from the corresponding author on reasonable request.
